# Self-Sensing Well Cement

**DOI:** 10.3390/ma14051235

**Published:** 2021-03-05

**Authors:** Kamila Gawel, Dawid Szewczyk, Pierre Rolf Cerasi

**Affiliations:** SINTEF Industry, SP Andersens vei 15b, 7031 Trondheim, Norway; dawid.szewczyk@sintef.no (D.S.); pierre.cerasi@sintef.no (P.R.C.)

**Keywords:** cement, carbon nanofibers, composite, resistivity, acoustic emission, self-sensing, unconfined compression

## Abstract

Chemical reactions with reservoir fluids and geology related in-situ stress changes may cause damages to cement sealing material in plugged and abandoned oil, gas and CO_2_ wells. To avoid leakages, a legitimate monitoring technique is needed that could allow for early warning in case such damages occur. In this paper, we test the utility of oil and gas well cement with a conductive filler in sensing stress changes. To this end, we have measured the resistance response of Portland G—oil and gas well cement with carbon nanofibers (CNF) to axial load during uniaxial compressive strength test. Simultaneously, the microseismicity data were collected. The resistance of the nanocomposite was measured using two-point method in the direction of loading. The resistance changes were correlated with acoustic emission events. A total of four different material response regions were distinguished and the resistivity and acoustic emission changes in these regions were described. Our results suggest that the two complementary methods, i.e., acoustic emission and resistance measurements, can be used for sensing stress state in materials including well cement/CNF composites. The results suggest that the well cement/CNF composites can be a good candidate material to be used as a transducer sensing changes in stress state in, e.g., well plugs up to material failure.

## 1. Introduction

The lifespan of all wells used in oil and gas (O & G) industry, including CO_2_ storage, ends with permanent plugging and abandonment (P&A). The plugging upon abandonment aims on eternal sealing of the well to prevent leakage of the remaining hydrocarbons and water from the reservoir to the environment [1]. Various countries have different requirements for P&A operations which may result in different lifetimes of plugs [1]. To convince the authorities and the society about the legitimate execution of P&A operation proper monitoring techniques are needed. Currently used techniques do not differ from typical methods used during exploration phase and include, among others, electromagnetic methods, seismic imaging, and pressure testing. Most of the monitoring is being done at the early stage of the P&A and, in most cases, no long-term follow-ups take place, which is partially due to the removal of all operational equipment from well prior to P&A cementing. This implies that the responsible authorities have to use only partial data while judging on whether or not installed well plugs are fulfilling their role and they have no control over when eventually the plugs fail to be a proper barrier due to chemical or mechanical degradation. The chemical degradation can result from interaction of the plug with reservoir fluids [2,3], while the mechanical failure can result from e.g., subsidence, shear displacement along discontinuities (e.g., rock interfaces, fractures) or change of in-situ stresses. Failure of a cement plug is considered as one of the main factors responsible for possible leakages into the upper overburden layers during P&A [1,4]. Therefore, continuous in-situ monitoring of well barrier materials could allow for early-stage warning of any loss of their sealing capacity and intervention before any leakage occurs. Chemical or mechanical degradation processes could possibly be detected by incorporating into the cement plug a sensor whose transducer is sensitive to both stress and changes in chemical environment.

Besides P&A, there could be a huge benefit in being able to monitor the state of stress of a well’s cement sheath also during its productive lifetime, as a production or injection well. It can be imagined that one could gather information on stress changes outside of the well, either due to thermal stress development accompanying temperature gradients from fluid injection, or as a consequence of depletion of the reservoir zone. In particular, the monitoring could give early warning of well shearing or even of fracturing in the near-well area and changes in fluid pressure there. Additionally, in the case of unconventional hydrocarbons, where fracturing is an essential operation to stimulate productivity from tight shales or sands, unwanted fracture propagation towards the cement sheath would be detected by this last.

It has been shown that cement materials with well dispersed conductive fillers such as e.g., metal fibers, graphite powder, carbon nanofibers, carbon nanotubes show stress/strain and mechanical damage sensitivity [5,6,7,8,9,10,11,12,13]. Owing to this property the composite materials are also known as self-monitoring, intrinsically smart, pressure-sensitive or piezoresistive materials [14,15,16]. Piezoresistivity is a physical property of materials defined as the change of the electrical resistivity upon exposure to stress. The physical mechanism underpinning this phenomenon is associated with the connectivity between the conductive particles. When a stress is applied to the material with embedded electrically conductive fillers, the inter-particle distance in the filler decreases, and new conductive paths are created. The closer the conductive particles are, the more interparticle connections are created, thus the resistivity of the material decreases. Provided there is no plastic deformation or microdamage in the material, upon unloading the composite material returns to its primary state and the initial resistivity is recovered. The response of the material to stress takes place only above a critical concentration of conductive particles called percolation threshold. This phenomenon is well explained by the percolation theory [17,18]. Due to their stress sensitivity, piezoelectric cement materials are considered as excellent sensors in structural health monitoring of reinforced concrete structures [6,19] and traffic monitoring [20,21,22]. Above certain strain the observed resistivity changes become irreversible upon unloading [15] and this effect is utilized to damage detection and localization [14,23,24].

In the work presented in this paper we aim at utilizing the stress sensitivity of cement composite materials containing carbon nanofibers in the sensing of stress changes that may occur in a well as a result of e.g., subsidence, fracture reactivation, hydraulic stimulation of reservoirs, etc. To this end, we test resistance changes of well cement (Potrland G, Norcem, Norway) with carbon nanofibers upon continuous, stepwise axial load increase up to material failure. We correlate the observed changes with a complementary acoustic emission method.

## 2. Materials and Methods

### 2.1. Materials

Portland cement class G from Norcem Heidelberg Cement Group was used. Portland cement class G is typically used to seal off oil and gas wells. Carbon nanofibers (CNFs) Pyrograf PR-19 XT-LHT were supplied by Applied Sciences Inc (Cedarville, OH, USA). An average length of nanofibers is in the range of 50–200 μm and diameter is about 150 nm. The received fibers are heat treated at 1500 °C to carbonize chemically vapor deposited carbon compounds present on their surfaces. The heat treatment, according to the supplier, provides the highest electrical conductivity in nanocomposite materials. The cement/CNF slurry was prepared using deionized water. MasterGlenium SKY 899 (BASF, Berlin, Germany) superplasticizer polymer was used as dispersing agent for carbon nanofibers.

### 2.2. Sample Preparation

**Figure 1:**
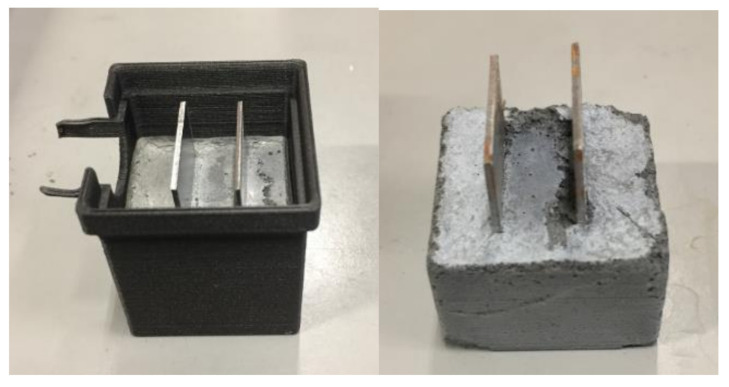
Photography of sample in the 3D printed mold and sample removed from the mold.

First, CNFs were dispersed in water/dispersing agent (MasterGlenium SKY 899) solution. CNFs are hydrophobic particles and they require application of dispersing agent to improve the dispersion in cement slurry. The CNF to dispersant weight ratio used in this work was 5:2. CNF dispersion was mixed with cement and additional water to yield water to cement ratio 0.58. The CNF/cement weight ratio was 0.03 for all samples. The cement/CNF slurry was hand mixed in 3 min before it was molded in a 3D printed cubic forms with dimensions of around 3 cm × 3 cm × 3 cm. Two steel plates 2 cm × 3 cm were inserted into the slurry with a 1 cm thick separator placed between them. The plates act as metal connectors between the cement/CNF material and the ohmmeter. Figure 1 shows photography of a sample in 3D printed mold with embedded connectors after 24 h of hardening. After 1 day of hardening at room conditions, samples were placed in sealed plastic bags to prevent water evaporation. After four months of further hardening, the experiments with uniaxial compression were performed.

### 2.3. X-ray Micro-Computed Tomography (µ-CT)

X-ray micro-computed tomography (µ-CT) was performed in order to find out whether CNFs were homogeneously distributed within the whole sample. It was performed using an industrial CT scanner (XT H 225 ST, Nikon Metrology, Geldenaaksebaan 329 Leuven, Belgium) that has been operated at 210 kV and with a current of 155 μA. A tin filter was used. The raw CT data were reconstructed into cross-sectional slices. The resolution of the CT images was around 30 mm/1310 pixel = 0.02 mm/pixel.

### 2.4. Uniaxial Compression Experiments

**Figure 2:**
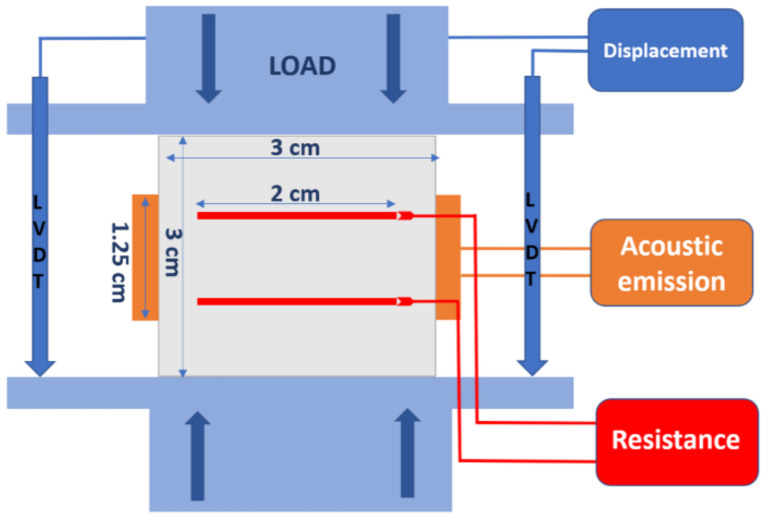
Schematic representation of experimental setup (not to scale) with indicated sizes of sample, electrical connectors and acoustic sensors.

In order to ensure surface parallelism, samples were grinded prior to testing. The load was applied via MTS electromechanical loading frame. The strains were measured globally by set of 3 linear variable differential transducers (LVDT’s) mounted at the top and bottom endplates (see Figure 2). To avoid the polarization effects the UCS tests were not following the typical ISO standards. Instead, the load was applied in step wise manner with pauses every 500 N. The pause times was necessary to perform resistance measurements. Measured force (***F***) and displacement (Δ***z***) were changed into the stress and axial strain with the use of typical formulas i.e.,:

(1) $$\sigma =\frac{F}{A}$$

(2) $$\varepsilon_{Z}=\frac{∆z}{z}$$

where ***σ*** stands for stress, ***ε_Z_*** is the strain in axial direction, whereas ***A*** is the cross-section area.

### 2.5. Resistance Measurements

Changes in sample resistance upon application of uniaxial load were followed using 117 True RMS multimeter (Fluke, Everett, WA, USA). The multimeter measures the resistance (*R*) using a DC current of 0.5 mA or less (depending on range), and an open voltage of no more than 4 V. The resistance was measured in the direction of load, i.e., electrical connectors were perpendicular to the load vector (See Figure 2). Resistance (*R*) between two connectors (two-point method) was measured for each load step. The bulk resistivity (ρ, or volume resistivity) of materials can be calculated according to the following formula:

(3) ρ=RA/l

where: *R* is the electrical resistance measured between connectors; *A* is surface area of connector; *l* is a distance between connectors.

Both the connector surface area in contact with conductive composite (380 mm^2^) as well as the distance between the connectors (10 mm) were identical for S1 and S2; thus, the bulk resistivity values are proportional to resistance with the same proportionality coefficient (*A/l* = 0.038 m) for all samples.

Due to polarization, the measured resistivity increases with time. To minimize the contribution of polarization, the resistivity was always measured a couple of minutes after the conditions (load) was changed. It is known that incremental increase of resistance due to polarity diminishes with time and is mainly in the first 50 s [25]. This means that the resistivity increase that is attributed to load changes in this work has little contribution from polarization.

### 2.6. Acoustic Emission

Acoustic emission events were acquired using AMSY-5 system (Vallen Systeme Gmbh, Icking, Germany). The low cut off filter was set to 33.7 dB. Two independent transducers Panametrics V103-RM (1 MHz, P-wave, 0.5 in diameter)(Baker Hughes, Houston, TX, USA) were mounted in the middle of sample on the sample walls perpendicular to the loading axis.

## 3. Results

**Figure 3:**
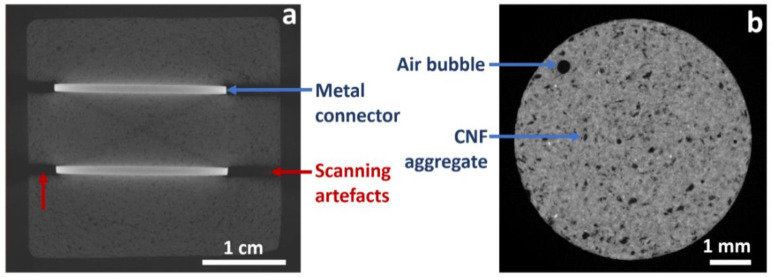
X-ray tomography cross section through the specimen with indicated metal connectors, and scanning artefacts originating from beam hardening effect (**a**) and sample of the same cement materials scanned at higher resolution (**b**).

The X-ray tomography imaging of the specimen, which was identical to the two tested specimens (S1, S2), was performed and the tomography cross section through the sample is presented in Figure 3a. The tomography distinguishes objects based on density difference. High density material is correlated with high X-ray attenuation and, thus, is represented by high intensity objects on the tomography images. In the tomography scan in Figure 3a, the metal connectors appear as bright rectangles. The very dark strikes, visible on the pictures as an extension of very bright metal connectors, are scanning artefacts that are associated with beam hardening and should be neglected. The darker cross visible between connectors is the same type of scanning artefact. Nevertheless, it is clear from the tomography imaging that the cement/CNF material is rather homogeneous at the millimeter scale. The darker spots within the cement, present at sub-millimeter scale, may indicate the presence of micro-sized CNF aggregates and/or air inclusions. Indeed, the tomography image taken from the same batch of cement/CNF material but imaged at smaller field of view (5.5 mm) and without metal objects (Figure 3b) indicates that there are two types of inhomogeneities present in the material. These are: (1) regular shape spherical air bubbles smaller than 500 μm in size; (2) irregularly shaped CNF aggregates, typically smaller than 150 μm.

**Figure 4:**
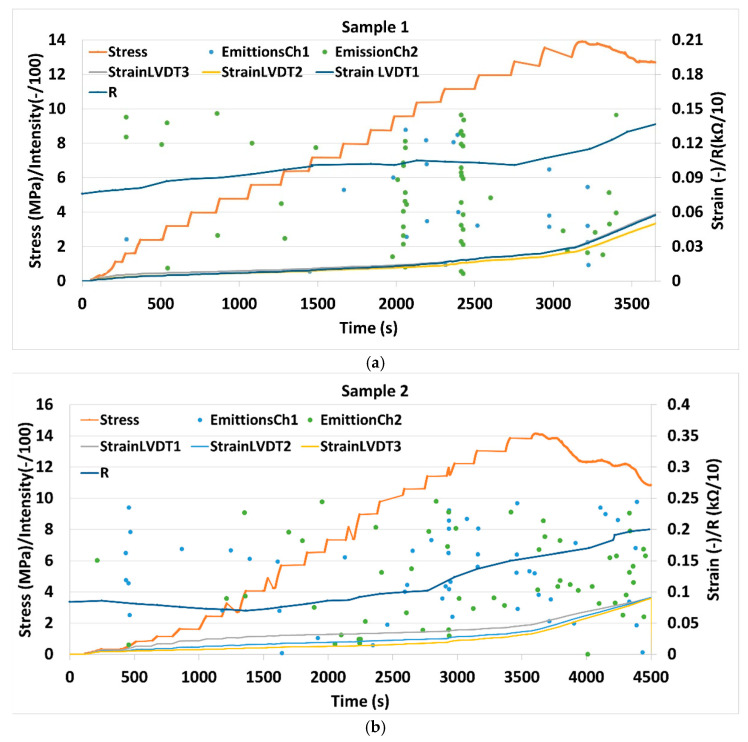
Stress (orange), strain measured by three different linear variable differential transformer sensors (LVDTs) (light blue, yellow, violet), resistivity (dark blue) and the intensity of acoustic emission events (blue and green dots) vs. time during uniaxial compression of specimens S1 (**a**) and S2 (**b**).

Changes in both resistivity as well as acoustic emission events were registered for S1 and S2 specimens upon application of mechanical load. Figure 4 shows changes in sample resistance, strains calculated from the three linear variable differential transformer sensors (LVDTs) during applied uniaxial compressive loading, in the whole range of stresses up to mechanical failure.

**Figure 5:**
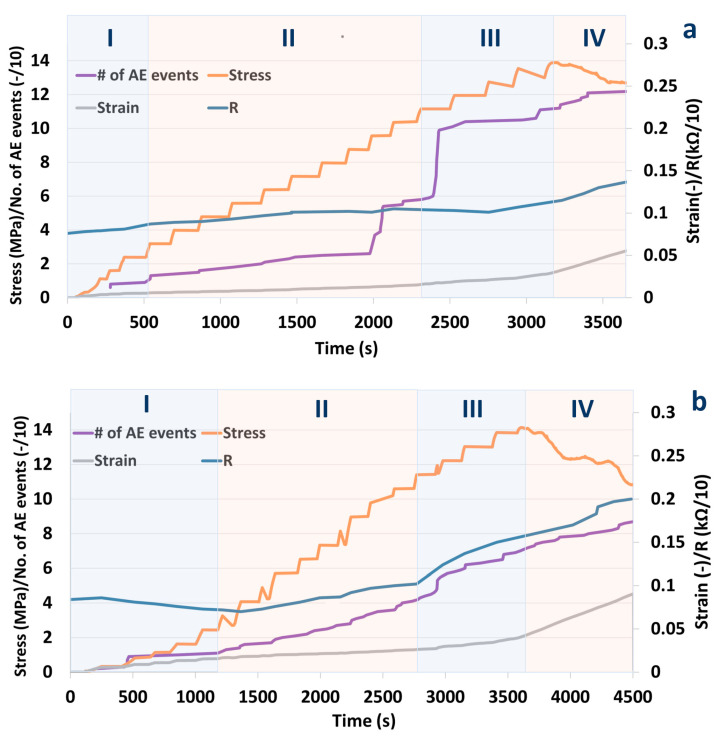
Evolution of stress, strain, resistivity, and the cumulative number of acoustic emission events during uniaxial compression of Sample 1 (**a**) and Sample 2 (**b**).

Figure 5 shows the evolution of the accumulated number of acoustic events along with resistance, strain and stress applied to the specimens. Before the load was applied, the resistance of sample S1 was measured to be 780 Ω, while for S2, it was 840 Ω. This corresponds to a bulk resistivity of 29.6 Ωm and 31.9 Ωm for S1 and S2, respectively (for details see Materials and Methods section). Figure 3 shows good reproducibility of the results for two different specimens. A total of four different characteristic phases can be distinguished in the plot: Phase I from 0 to load up to around 2.3 MPa where resistivity is relatively constant, or even decreases and no acoustic events are registered. Phase II stretches from a stress of 2.3 MPa up to around 10 MPa. In this phase, resistivity is slowly increasing and so is the number of acoustic events. In phase III (10-14 MPa) the number of acoustic events is significantly increasing along with material resistance. The failure of both samples occurred at around 14 MPa. At failure, the S1 and S2 resistances were 1150 Ω and 1700 Ω, respectively. The higher resistivity at failure for sample S2 coincides with the larger number of acoustic events observed in this sample. After failure, in phase IV, the resistivity and the number of acoustic events continued to increase.

**Figure 6:**
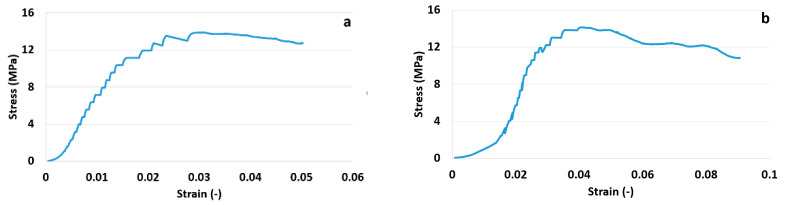
Stress vs strain for Sample 1 (**a**) and Sample 2 (**b**).

Figure 6 shows a standard representation in the stress–strain space of the two tests. The tests were conducted under controlled axial strain conditions, in a step-wise manner. The obtained axial stress as a function of axial strain in Figure 6 shows typical shapes for ductile cement: a first non-linear part where stiffness slowly increases, a linear part followed by a yield point and finally a peak after which stress decreases slowly. The slope of the final post-peak part of the curve differentiates ductile vs. brittle fracturing behaviour. The more the curve remains at higher stress values, close to peak value, the more the cement behaves as a ductile solid.

## 4. Discussion

When the cement/CNF composites are subjected to compressive loading, at the beginning, the electrical resistance is constant or it declines (phase I) and very few acoustic emission events are registered. This can be clearly seen in traditional stress-strain representation, where apparent hardening occurs in phase I (meaning that as deformation increases, stress accelerates resulting in a stiffening of the material). This initial phase is typical of UCS tests, where lateral support of the tested plug is inexistant and inevitable settling and microfracture closure occur very early in the test. Up to 2.3 MPa, the cement/CNF material probably undergoes elastic deformation or at least no micro-fractures are yet induced [26]. The resistance decrease, as a response to load in this region observed by many authors [9,12,13,19,21,22,27] has been ascribed to the formation of additional electrical connections between fibers due to compaction of the material [16]. There exists controversy in literature around reversibility of resistance after unloading. Both complete reversal of resistance [6] as well as irreversible, gradual increase in resistance [15] have been observed upon loading-unloading cycles in the elastic regime. Wen Sihai et al. [15] reports small irreversible resistivity changes after cycles in the elastic regime in the absence of irreversible strain. This, according to the authors, indicates minor damages occurring already in the elastic regime that could be, e.g., breakage of tubes/fibers. To avoid wear damages similar to those reported by Wen Sihai et al., associated with cycling in our experiments, loading was increased stepwise and continuously. Thus, wear damages are eliminated such that the resistivity changes are instead associated with continuous load application only.

Phase II translates to a straight line in stress–strain domain (Figure 6); however, this does not guarantee elasticity, as, often, when one incorporates an unloading loop even below half-peak stress values, hysteresis is observed in strain [28]. This means that deformation, even in the linear plot portion, is a combination of elastic (recoverable) and plastic (permanent) parts. There is, however, a well-defined yield in the same plot, where phase III starts, which indicates a transition into full plasticity. For geomaterials, this indicates coalescence of fractures [29] into what will become a sample-traversing shear band and bifurcation towards localized shear deformation. This is supported by the larger jumps in acoustic emission just before and at the beginning of phase III. This further increase in load, in the developing plastic deformation regime (phase II and III), is associated with a gradual increase in resistivity. According to Wen Sihai et al. [15], the resistivity changes in the plastic deformation regime (with irreversible strain) are associated with irreversible major damages occurring in the material. Frequent acoustic emission events in this region indeed indicate micro fracturing.

The following mechanism explaining resistance changes in the elastic and plastic regimes has been proposed [16]: compaction causes carbon fibers to approach each other. This improves the conductivity within the conductive network inside the cement. The initiation of new cracks leads to damage and reconstruction of the conductive network while the extension of cracks result in breakdown of the conductive network. Depending on which of the two mechanisms is dominating, either increase or decrease in resistivity is observed. Apparently, in the elastic region (phase I), crack compaction and the constitution of new electrical connections dominate while in the plastic region (phase II and III) crack initiation, propagation and degradation of electrical connectivity are dominating.

The results for the two similar samples Sample 1 and Sample 2 were qualitatively reproducible. However, resistance and bulk resistivities of the S1 and S2 specimens increased by 47 and 102%, respectively, compared to the state before the application of mechanical load up to the mechanical failure. This quantitative difference can most likely be ascribed to the stochastic nature of fracture growth and propagation. Additionally, slight variations in mixing of the cement powder, the presence of inevitable air bubbles and different distribution of fibers in the slurry contribute to structural differences between the two plugs. 

In phase IV, continued acoustic emission and resistivity increase probably originate from sliding of the two plug parts on the shear plane defined by the failure shear fracture after peak stress. It would be interesting to see whether localization of the acoustic energy coincides with the main fracture plane. Had the cement plug still been structurally intact, albeit with micro-cracks, a reduction below peak stress should not have resulted in new acoustic events, as predicted by the Kaiser effect [30]. This will however be a topic for a separate study.

## 5. Implication for Stress-State Sensing in Oil, Gas and CO_2_ Wells

A self-sensing well cement with carbon nanofibers can possibly be suitable as a permanent monitoring implement, sensing changes in the mechanical environment in wells and well plugs. If proven as a suitable technique at field scale, with the added technical and logistical problems of delivering power to the measuring sensors over time and distance, and conveying the measured signal back to surface, this cement additive could give early warning for any potentially well-damaging evolution of stress conditions in the near-well area. This would significantly reduce the need for and cost of workover interventions. However, the performance of the transducer has first to be optimized.

By far the most important undertaking would be to improve the quantitative reproducibility. This can likely be done by reducing microsized inhomogeneities in the material which can likely be achieved by reducing the amount of carbon- based conductive fillers in the material. It has been shown that materials with very low content of conductive fillers (below percolation threshold) can sense stress/strain changes [15] in cement materials. 

It would also be important to get a better understanding about the cement/CNF material response to pore pressure changes, as well as true-triaxial compression (different values for all three principal stress components). 

There are several methods that can be used to monitor resistance changes. In addition to the most common direct measurement methods, i.e., direct two-point (used in this paper) and four-point resistance measurements methods there are: controlled source electromagnetics (CSEM), electrical resistivity tomography (ERT), and magnetometric resistivity (MMR) methods that can be utilized [31,32,33,34].

## 6. Conclusions

- Portland G well cement with an embedded conductive filler was shown to be sensitive to mechanical load.
  The resistivity changes were large enough to be measured with the two-point resistance measurement method.
- A decrease in resistivity was observed in the elastic deformation region, while in the plastic deformation region, a significant increase in resistivity accompanied with a large number of acoustic events were observed.
- The resistivity increase continued beyond the failure threshold and so did the number of acoustic events.
- Comparison of the results for two similar samples suggest that results are qualitatively reproducible. However, the magnitudes of resistivity changes were different for the two analyzed samples. The quantitative discrepancy has been ascribed to the stochastic nature of fracture growth and propagation, strengthened by the presence of micro-sized inhomogeneities (nanofiber aggregates).
- The self-sensing well cement with carbon nanofibers can possibly be suitable as a device sensing changes in the mechanical environment in wells/well plugs. However, the performance of the transducer has to be optimized, the quantitative reproducibility has to be improved and the mutual response to pore pressure as well as true-triaxial compression have to be defined.
- Engineering cement/CNF composite weight and mechanical/elastic properties by adding heavy-weight hematite and barite or light-weight polymer microspheres could perhaps allow for broadening of the application range for cement/CNF materials in the future. Thus, describing how these additives affect resistivity and resistivity changes in response to load would be needed.

## Data Availability

The data presented in this study are available on request from the corresponding author.
